# Supplementary material for: A scoping review protocol on brain PaCO2 levels at altitude
Source: PLoS One. 2025 Jan 14;20(1):e0316608. doi: 10.1371/journal.pone.0316608 (PMC11731867; doi:10.1371/journal.pone.0316608)
Supplement: S1 Appendix — (DOCX) [file pone.0316608.s002.docx]

# Appendix 1

***PRESS Guideline* — Search Submission & Peer Review Assessment**

**SEARCH SUBMISSION: THIS SECTION TO BE FILLED IN BY THE SEARCHER**

| Searcher: RS |  |
| --- | --- |
| Date submitted: June 21, 2024 | **Date requested by:** *[Maximum = 5 working days]* |

**Systematic Review Title:**

| **A scoping review protocol on brain PCO_2_ levels at altitude** |
| --- |

This search strategy is …

| x | My PRIMARY (core) database strategy — First time submitting a strategy for search question and database |
| --- | --- |
|  | My PRIMARY (core) strategy — Follow-up review NOT the first time submitting a strategy for search question and database. If this is a response to peer review, itemize the changes made to the review suggestions |
|  | SECONDARY search strategy— First time submitting a strategy for search question and database |
|  | SECONDARY search strategy — NOT the first time submitting a strategy for search question and database. If  this is a response to peer review, itemize the changes made to the review suggestions |

**Database**

(i.e., MEDLINE,CINAHL…): *[mandatory]*

| Medline |
| --- |

**Interface**

(i.e., Ovid, EBSCO…): *[mandatory]*

| Ovid |
| --- |

**Research Question**

(Describe the purpose of the search) *[mandatory]*

| Does blood PaCO2 change acutely with exposure to high altitudes in humans, and if so, does this occur at cruising altitudes used in aeromedical transportation? |
| --- |

**PICO Format**

(Outline the PICOs for your question — i.e., Patient, Intervention, Comparison, Outcome, and Study Design — as applicable)

| **P** | All patients |
| --- | --- |
| **I** | PACO2 |
| **C** | High altitudes |
| **O** |  |
| **S** |  |

**Inclusion Criteria**

(List criteria such as age groups, study designs, etc., to be included) *[optional]*

|  |
| --- |

**Exclusion Criteria**

(List criteria such as study designs, date limits, etc., to be excluded) *[optional]*

|  |
| --- |

**Was a search filter applied?**

Yes: □ No: x

**If YES, which one(s) (e.g., Cochrane RCT filter, PubMed Clinical Queries filter)? Provide the source if this is a published filter.** *[mandatory if YES to previous question* — *textbox]*

|  |
| --- |

Other notes or comments you feel would be useful for the peer reviewer? ***[optional]***

|  |
| --- |

Please copy and paste your search strategy here, exactly as run, including the number of hits per line. ***[mandatory]***

Ovid MEDLINE(R) ALL <1946 to June 19, 2024>

1 Carbon Dioxide/bl 24054

2 paco2.tw,kf. 9578

3 (partial pressure* adj5 (arter* or blood or serum) adj5 carbon dioxide).tw,kf. 1502

4 (partial pressure* adj3 co2).tw,kf. 1862

5 (Arterial* adj5 carbon dioxide adj5 (concentration* or tension*)).tw,kf. 1630

6 (partial pressure adj4 carbon dioxide).tw,kf. 3314

7 or/1-6 35207

8 aircraft/ 10038

9 altitude/ 20671

10 altitude*.tw,kf. 33949

11 helicopter*.tw,kf. 4546

12 or/8-11 52101

13 7 and 12 625

14 exp animals/ not humans/ 5233406

15 13 not 14 491

**PEER REVIEW ASSESSMENT: THIS SECTION TO BE FILLED IN BY THE REVIEWER**

| **Reviewer**  **BD** |  | **Date**  **June 24, 2024** |
| --- | --- | --- |

| 1. **TRANSLATION** |
| --- |

| A -­‐No revisions | x |
| --- | --- |
| B -­‐ Revision(s) suggested | ☐ |
| C -­‐ Revision(s) required | ☐ |

If “B” or “C,” please provide an explanation or example:

|  |
| --- |

**2. BOOLEAN AND PROXIMITY OPERATORS**

| A -­‐No revisions | x |
| --- | --- |
| B -­‐ Revision(s) suggested | ☐ |
| C -­‐ Revision(s) required | ☐ |

If “B” or “C,” please provide an explanation or example:

|  |
| --- |

**3. SUBJECT HEADINGS**

| A -­‐No revisions | x |
| --- | --- |
| B -­‐ Revision(s) suggested | ☐ |
| C -­‐ Revision(s) required | ☐ |

If “B” or “C,” please provide an explanation or example:

|  |
| --- |

**4. TEXT WORD SEARCHING**

| A -­‐No revisions | x |
| --- | --- |
| B -­‐ Revision(s)suggested | ☐ |
| C -­‐ Revision(s) required | ☐ |

If “B” or “C,” please provide an explanation or example:

|  |
| --- |

**5. SPELLING, SYNTAX, AND LINE NUMBERS**

| A -­‐No revisions | x |
| --- | --- |
| B -­‐ Revision(s)suggested | ☐ |
| C -­‐ Revision(s) required | ☐ |

If “B” or “C,” please provide an explanation or example:

|  |
| --- |

**6. LIMITS AND FILTERS**

| A -­‐No revisions | x |
| --- | --- |
| B -­‐ Revision(s) suggested | ☐ |
| C -­‐ Revision(s) required | ☐ |

If “B” or “C,” please provide an explanation or example:

|  |
| --- |

OVERALL EVALUATION (Note: If one or more “revision required” is noted above, the response below must be “revisions required”.)

| A -­‐No revisions | x |
| --- | --- |
| B -­‐ Revision(s) suggested | ☐ |
| C -­‐ Revision(s) required | ☐ |

Additional comments:

|  |
| --- |
