# Supplementary material for: A scoping review protocol on brain PaCO2 levels at altitude
Source: PLoS One. 2025 Jan 14;20(1):e0316608. doi: 10.1371/journal.pone.0316608 (PMC11731867; doi:10.1371/journal.pone.0316608)
Supplement: Attachment — Submitted filename: Response to Reviewers.docx [file pone.0316608.s003.docx]

**Response to Reviewers:**

**Reviewer 1:**

**Comment 1: Helicopter cabins are usually not pressurized. Since cabin pressure is not considered an inclusion criteria factor, the authors should include a short discussion on how cabin pressure may affect the PaCO2.**

**Response:** This is an excellent point. Although rotary wing transfers are the most commonly used airframe for aeromedical transfers and are often unpressurized, fixed wing transport at higher altitudes is required for longer distances; fixed wing cabins for medical transfer are invariably pressurized, often to an equivalent altitude 2,000-8,000 feet. We have included the following in our concepts section under inclusion criteria: “**Higher cabin pressure is favoured from a medical perspective for the patient since it creates a relatively less hypoxic hypobaric environment.**” The trade-off is that a pressurized cabin will increase fuel usage and on some flight distances, result in longer flight times due to the need for refueling. We have not included cabin pressure as an inclusion criteria since we would like to first scope all available literature in the field to see if altitude affects PaCO_2_, and since cabins are normally pressurized to over 1000 feet. If there is a pressurized cabin system, we will note in our review the equivalent altitude to which the cabin was pressurized.

**Comment 2: The authors mention in lines 173-175 that the current protocol may have a potential limitation in that the number of related studies may be small. Do the authors have an estimated number of literatures before and after the screening?**

**Response**: Based on the initial search, we have found 491 articles prior to screening. We are not yet able to estimate the number of studies after screening, as we are awaiting approval of the final protocol before screening to maintain methodological rigor.

**Comment 3:** **Please provide a discussion session that expands on the current “Strengths and Limitations” and briefly elaborates on factors like high altitude exposure format (helicopter, airliners, mountain climbing, chamber), exposure time, etc, that could complicate data interpretation.**

**Response**: We have elaborated on the “Strengths and Limitations” section. The following was added to the protocol: “**It is unclear if altitude of aeromedical transportation would affect the rate of ischemic burden. If there is evidence that suggests that PaCO_2_ is impacted at cruising altitudes of aeromedical transport, then it opens the door to further studies in this area**… **Our preliminary search result yielded articles with various formats of high altitude exposure such as air transport, mountain climbing, and residing at high altitudes. One way we aim to address this weakness and make it more applicable to our clinical context is to ensure that PaCO_2_ was measured in at least two altitudes. Depending on the final list of included articles, it is not known if there will be sufficient evidence to comment on how brief exposures to high altitude impacts PaCO2. However, this could also in turn open the door to future studies to fill the potential knowledge gap**.”

**Comment 4: Keep the PaCO2 acronym consistent.**

**Response**: Thank you for bringing this to our attention. This has been corrected.

**Reviewer 2:**

**Comment 1: Exclusion Criteria: The authors indicate that they will exclude studies involving participants under 18 years of age. However, it is unclear why this exclusion criterion is necessary for their review.**

Response: The majority of the Canadian Stroke Guidelines are for patients aged 18 and older. Ultimately the goal of this study is to provide future direction to improve our adult stroke care, which is the population who we serve clinically. Additionally, it is unclear if neonates and/or children would have the same hypoxic drive to breathe at altitude as in adults. But we appreciate the comment and agree after this initial review, our team will consider a similar assessment in the pediatric population.

**Comment 2: Measurement of PaCO₂: The authors specifically aim to include studies that report arterial partial pressure of CO₂ (PaCO₂) at two different altitudes. Since studies may measure PaCO₂ either directly (e.g., via arterial blood gas) or indirectly, the authors should clarify their criteria regarding acceptable methods for PaCO₂ measurement**.

**Response**: Very good point – we have now included the following statement in our inclusion criteria: “**We will include studies that comment on PaCO_2_ measured both directly via arterial blood gas and indirectly**.”
